# Quality of life and its contributors among adults with late-onset Pompe disease in China

**DOI:** 10.1186/s13023-021-01836-y

**Published:** 2021-05-01

**Authors:** Shanquan Chen, Jingxuan Wang, Jianfeng Zhu, Roger Yat-Nork Chung, Dong Dong

**Affiliations:** 1 The School of Clinical Medicine, University of Cambridge, Cambridge, UK; 2 JC School of Public Health and Primary Care, Faculty of Medicine, 4/F School of Public Health, Prince of Wales Hospital, The Chinese University of Hong Kong, Hong Kong SAR, China; 3 School of Social Development and Public Policy, Fudan University, Shanghai, China; 4 CUHK Institute of Health Equity, The Chinese University of Hong Kong, Hong Kong SAR, China; 5 Shenzhen Research Institute, The Chinese University of Hong Kong, Shenzhen, Guangdong China

**Keywords:** Quality of life, Pompe disease, Rare disease, Cross-sectional survey, China

## Abstract

**Background:**

Pompe disease (PD) is a rare inherited disorder caused by the deficiency of acid-α glucosidase, which leads to the impairment of organ and tissue functions and causes disabilities. As the first national survey on patients with late-onset PD (LOPD) in China, this study investigated the quality of life (QOL) of adult patients with LOPD in China and explored its contributors.

**Methods:**

Data were derived from a nation-based, cross-sectional, self-response survey on rare diseases (RDs) in early 2018. Answers from 68 adult Chinese patients with LOPD were used for data analysis. QOL was measured using the World Health Organization Quality of Life: Brief Version. Covariates included age, gender, education, employment, reliance on assistive devices, medication history, social support, and disease economic burden. Data were analyzed using linear regression in R.

**Results:**

For adult patients with LOPD, the average scores and standard deviations (SD) of the four dimensions of QOL were physical health = 33.77 (SD = 18.28), psychological health = 43.81 (SD = 21.70), environmental health = 39.43 (SD = 16.93), and social relationship = 46.20 (SD = 19.76); the scoring for each dimension was evaluated on a scale of 0 to 100. At the significance level of *p* < 0.05, with increasing age, the patients experienced a significant decrease in physical health QOL (β = − 0.75) and environmental health QOL (β = − 0.79). Those who relied heavily on assistive devices had lower perceived physical health (β = − 17.8), psychological health (β = − 22.76), environmental health (β = − 17.8), and social relationships (β = − 22.12) than those who did not. A one-unit increase in the amount of social support, as a form of social interaction, led to a significant increase in physical health (β = 0.28), psychological health (β = 0.71), environmental health (β = 0.72), and social relationships (β = 0.70).

**Conclusion:**

Adult Chinese patients with LOPD had a lower physical health and QOL compared to their counterparts with other RDs. Being employed was found to affect the QOL of adult Chinese patients with LOPD in almost all dimensions. Encouraging adult Chinese patients with LOPD to be socially active and help them become more involved in social life might improve their QOL.

## Background

Pompe disease (PD), also known as glycogen storage disease type II, is an inherited disorder caused by the deficiency of acid-α glucosidase (GAA) [1]. PD is generally classified into two forms: (1) infantile-onset PD (IOPD) when the disease is characterized by cardiomyopathy, respiratory insufficiency, and severe muscle hypotonia, and presents during the first year of life, and (2) late-onset PD (LOPD), which encompasses the remaining patients with PD who can present at less than 12 months without cardiomyopathy or present after 12 months [2]. In patients with PD, the accumulation of glycogen in the skeletal muscles, heart, liver, and nervous system causes a decline in their quality of life (QOL) and finally leads to disabilities or even death [3, 4].

The prevalence of PD is related to ethnicity or the geographic area studied [5]. According to a recent study in Taiwan, the birth prevalence of IOPD in Taiwan is 1 in 52,000, and of LOPD is about 1 in 25,000 [6]. Since mainland China does not have its own official estimates, using the Taiwanese birth prevalence as a proxy, and the number of new births in China in 2018 (15,230,000), the number of individuals born with IOPD in China in 2018 can be estimated to be at 293 (52,000/15,230,000), and individuals with LOPD at 609 (25,000/15,230,000). Although having a relatively small population size, PD is one of the 5% of all rare diseases (RDs) that have certain treatments, most of which are labeled orphan drugs that often imply a small consumer market, a high price, and a heavy financial burden for their patients. As a result, the quality of life in patients with PD and those associated with them is of great concern.

International studies found that LOPD is negatively associated to QOL in physical health domains, while inconsistent evidence was found for QOL in mental health and functional-emotional domains [7–9]. Despite the negative consequences of PD on physical health, ongoing and chronic illness stressors may induce emotional and cognitive adjustments to the disease. Some patients may handle the psychological burden of PD relatively well, while others may not [10]. Variations in the QOL of patients with LOPD were observed across countries and ethnicities. For instance, the International Pompe Survey found that among 210 patients with LOPD in five countries, patients in the United Kingdom had significantly lower scores in vitality but much higher scores in perceived general health than American, Australian, German, and Dutch patients [8]; and in other dimensions, i.e., social and emotional functioning, Australians performed the worst among the five focal countries. Despite the emphasis on international and inter-racial variations, previous studies were mainly conducted in Europe and North America, while the situation in China has never been comprehensively reported.

Among the very few studies on the determinants of QOL in patients with PD, those in Western societies have emphasized enzyme replacement therapy (ERT) as a significant promoter of QOL, especially at the early stages of treatment. However, adverse infusion reactions have also been reported [11]. In China, it is estimated that the average annual cost of ERT for an adult PD patient is around RMB 3,000,000 (1 USD ~ 7 RMB), which is over four times the average annual household income for a Chinese family. Considering the low coverage rate of social and commercial insurance for ERT, Chinese patients with PD are fully exposed to the potentially catastrophic consequences of the disease. By the end of 2017, among the 61 patients with PD (including both IOPD and LOPD) who were registered to a national PD patient organization, only 12 had ever used the only available ERT in China, alglucosidase alfa. Among these 12, only four patients had used the medicine continuously, and none had used it up to the dosage approved by the National Medical Products Administration (i.e., 20 mg/kg every other week [12]. From 2014 to 2018, 14 child patients with PD passed away, and 11 of them had never used the drug due to unaffordability [12]. Considering the high cost and low coverage of ERT, the levels and determinants of QOL are of great concern in Chinese adult patients with LOPD. Therefore, the main objective of this study was two-fold: (1) to assess the QOL of adult patients with LOPD in China, and (2) to explore the social and economic factors that may contribute to it.

## Methods

### Sampling methods

The data used for this research were derived from a large, nation-based, cross-sectional survey among people affected by RDs in China in 2018 [13–15]. Since no complete sample frame of patients with RDs exists due to the fact that the epidemiological information of these people is largely unknown in China, a non-probability convenience sampling method was used to recruit participants in collaboration with a variety of national RD patient organizations, namely the Illness Challenge Foundation and its “29 + Alliance” for RD patient organizations, including the China Pompe Care Center (CPCC), the only national Pompe patient organization in China. The survey details were distributed via online and offline social networks. Individual patients also shared recruitment information.

### Procedure

The survey was conducted primarily online (www.wenjuan.com) to maximize accessibility to the dispersed population. It was approved by the Committee on the Use of Human and Animal Subjects in the Teaching and Research of Hong Kong Baptist University (No: FRG2/15-16/052). Although the survey was self-administered, previous studies have shown that this type of survey could yield a higher response rate than the physical mail survey and more accurate results than the telephone survey [16, 17]. Participants followed a link to the survey website. Informed consent was obtained from the participants before they were presented the questionnaire. At the beginning of the questionnaire, a series of questions were used to identify the target respondents of the survey (i.e., people affected by RDs in China). Patients under 18 were asked to end the survey and refer the survey link to their legal guardians. Main caregivers and patients were identified and assigned to one of the two versions of the questionnaire. The two versions covered the same measures, but the questions were formed differently to retrieve more accurate answers. The survey was conducted from January 1 to February 15, 2018.

### Patients with PD in this study

In total, 2007 valid responses were collected from the 2018 survey, which covered 94 distinctive types of RDs. The survey included responses from 92 patients with PD. Although small in number, the 14 patients with IOPD and 78 patients with LOPD were all patients with PD with a definitive clinical diagnosis and had registered with the CPCC by the time of the survey. In other words, these 92 patients comprised the entire population of patients with PD that could be reached in China by February 15, 2018. In comparison, a newly published article on the Pompe registry in China mentioned that it has only 78 registered patients [18]. Thus, our sample was, in fact, a complete set of all possible observations on Chinese patients with PDat the time of the survey.

Among the 78 surveyed patients with LOPD, 68 were adults and 10 were children. Since the scales we used to examine QOL and social support were personal measures, only adult patients could answer these questions by themselves. Hence, to maximize the reliability and accuracy of the answers, this study only included 68 adult patients.

### Measurements

#### Dependent variable: quality of life

Quality of life was measured by the World Health Organization Quality of Life: Brief Version (WHOQOL-BREF**)**, which was developed collaboratively by experts around the world and has been widely field-tested [19]. In an international survey of 11,830 adults from 23 countries, the WHOQOL group reported that WHOQOL-BREF was a reliable and cross-culturally valid assessment of QOL across all four domains [20]. According to the same research, the Cronbach’s alpha for the four domains in a Chinese sample was acceptable for the physical domain (0.82), the psychological domain (0.89), the social domain (0.76), and the environment domain (0.70). In addition, WHOQOL-BREF was extensively used in China to measure the QOL of older adults [21], patients with specific diseases [22–25], and patients with RDs [26]. The WHOQOL-BREF assesses individuals’ perceptions in the context of their culture and value systems and their personal goals, standards, and concerns. The instrument comprises 26 items that measure the following broad domains: physical, psychological, social relationships, and environment. The physical domain refers to the pain, energy, sleep, mobility, activities, medication, and work of a patient. The psychological domain emphasizes patients’ positive/negative feelings, cognitions, self-esteem, body image, and spirituality. The social domain evaluates personal relationships, and social and sex activities. The environmental domain focuses on patients’ subjective perceptions of safety, home environment, leisure, transportation, and health/social facilities [19, 20]. A guideline published by the WHO illustrated in detail the scores calculated for each domain [19]. The calculated maximum score is 100 in all domains, and scores are scaled in a positive direction (i.e., higher scores denote higher levels of quality of life). It has been noted that the cut-off for the WHOQOL-BREF varies among different groups of people. Elderly studies found a critical value of 60 as the best cut-off point [27]. Yet another study on urban residents in China revealed that the two cut-off standards for low QOL, “70% of the maximum score” and “1 SD below the mean”, produced very different results for the probable prevalence of low QOL in the population under investigation [28].

#### Independent variables

The independent variables in this study included sociodemographic variables (age, gender, education, employment status, and residence), medication history, disease economic burden, reliance on assistive devices, and social support.

Medication history was assessed by asking the respondents whether they received ERT with recombinant human GAA (rhGAA, Myozyme) during the past 12 months. Economic burden was measured in terms of catastrophic health expenditure (CHE). When the cost of treatment for a disease exceeds a certain percentage of income, the disease is considered to cause CHE for the patient. There is no commonly accepted criterion for defining CHE [29], and it varies from 10% of income [30, 31], or 10% of household consumption [32] to 40% of disposable income [33, 34]. In this study, we regarded out-of-pocket health expenditures in excess of 10% of annual family income as an indicator of CHE. Reliance on assistive devices is an important indicator of disability status, and was measured with patients’ self-reports on the degree to which they needed to rely on assistive devices in their daily lives. Social support was measured with the Chinese Mandarin version of the Medical Outcomes Study Social Support Survey (MOS-SSS-CM), which is a brief, multidimensional, self-administered instrument developed for patients with chronic conditions and has been validated previously among patients in mainland China [35]. The MOS-SSS-CM consists of 20 questions. One single item measured the structural support by asking “How many close friends and relatives (refers to people who you can get along with and talk about your concerns) do you have?”. The other 19 items measured functional support from four subscales (emotional/informational support, tangible support, affectionate support, and positive social interaction) [36].

### Data analysis

In this study, the QOL was analyzed as sub-dimensional scores rather than a total score in order to reveal more specific information. Descriptive analyses included means and standard deviations of the continuous variables and percentages of categorical data. Univariate analysis of QOL included a t-test for binary variables, ANOVA test for categorical variables with more than two categories, and the Pearson correlation test for continuous variables.

To help understand that a certain contributing factor in excess could reduce the QOL and vice versa, we conducted a multivariable linear regression. Variables with *p* < 0.1 in the univariate analysis were included in the linear regression for multivariable analysis. All statistical analyses were conducted using R software (version 3.5.0), including packages of *dplyr* (version 0.8.0.1) and stats (version 3.6.0). Statistical significance was defined as *p* < 0.05.

## Results

### Descriptive statistics

Table 1 summarizes the main variables investigated in this study. A total of 68 adult patients with LOPD were included in the study. To better outline the patients’ social, medical, and economic characteristics, we compared them with those of patients with other RDs who participated in the same cross-sectional survey.

**Table 1:** Basic descriptive statistics

		Adult patients with LOPD N = 68 %(n/N)/mean (SD)^†^	Adult patients with other RDs (Non-Pompe) N = 1067 %(n/N)/mean (SD)^†^	*p *value
Age (years)		30.00 (7.39)	35.71 (11.21)	**< 0.001**
Age at the onset of symptoms		20.68 (6.71)	22.06 (15.60)	0.148
Time between the onset of symptoms and first time to seek diagnosis		0.12 (4.73)	2.75 (6.28)	**< 0.001**
Time between the first time to seek diagnosis and being diagnosed		4.25 (5.03)	3.23 (6.25)	**< 0.001**
Gender (= male%)		47.06 (32/68)	46.20 (493/1,067)	0.991
Education (%)				
Primary school or lower (6 years of edu)		7.35 (5/68)	12.00 (128/1,067)	0.505
Middle school (9 years of edu)		22.06 (15/68)	21.84 (233/1,067)	
High school or above (12 years of edu)		70.59 (48/68)	66.17 (706/1,067)	
Employment (= yes%)		20.59 (14/68)	52.67 (562/1,067)	**< 0.001**
Catastrophic health expenditure (CHE) (= yes%)		67.21 (41/61)	70.03 (687/981)	0.748
Rural/Urban (= rural%)		35.29 (24/68)	24.55 (262/1,067)	0.067
Reliance on Assistive Devices (%)				
None		8.82 (6/68)	57.73 (616/1,067)	**< 0.001**
Some		50.00 (34/68)	27.09 (289/1,067)	
A lot		41.18 (28/68)	15.18 (162/1,067)	
Medicated (= yes% “used ERT in the past 12 months”)		10.29 (7/68)	–	–
Social support (out of 100 points)				
Tangible support		72.87 (15.72)	64.78 (20.43)	**< 0.001**
Emotional/informational support		55.70 (16.01)	54.86 (18.21)	0.680
Positive social interaction		52.65 (15.85)	53.72 (18.89)	0.594
Affectionate support		55.78 (17.35)	56.48 (20.10)	0.751
Structural support		5.63 (5.83)	5.62 (10.25)	0.990
Quality of life (out of 100 points)				
Physical health	33.77 (18.28)		48.22 (20.87)	**< 0.001**
Psychological health	43.81 (21.7)		43.25 (20.76)	0.837
Environmental health	39.43 (16.93)		42.70 (17.80)	0.127
Social relationships	46.20 (19.76)		48.56 (19.54)	0.342

Bold values indicate statistical significance was defined as* p* < 0.05

^†^SD refers to the standard deviation

“–” means ERT is not available or applicable to adult patients with other RDs under our survey

The average age of adult patients with LOPD was 30 years. The male-to-female ratio was around 1:1.1. About two-fifths of the participants lived in rural areas. More than 92% of them completed the nine-year mandatory education (or reached the level of middle school), while more than 70% completed 12 years of education (or reached the level of high school or above). Approximately one in five adult patients with LOPD is currently employed. Only 10% of the 68 patients used Myozyme in the past 12 months, and their average age was younger than that of non-users (27.9 years vs. 30.2 years). Nevertheless, nearly 70% of them suffered from CHE. Half of the adult patients with LOPD relied moderately on assistive devices, and around 40% relied heavily on them.

Compared with the 1067 adult patients with RDs other than PD, the 68 patients with LOPD were younger (30.0 years among patients with LOPD vs. 35.7 years among patients with other RDs), experienced a shorter period between the onset of symptoms and first time to seek diagnosis (0.12 years vs. 2.75 years), yet a longer period between the first time to seek diagnosis and finally being diagnosed (4.25 years vs. 3.23 years). Patients with LOPD are also less likely to be employed (20.6% vs. 52.7%) and more likely to rely on assistive devices (91.18% vs. 42.27%). The other factors, including gender, education, and CHE, were similar between the two groups.

Conversely, the perceived social support of adult patients with LOPD in China appears to be similar to that of other Chinese patients with RDs. For patients with LOPD, the average scores of the four dimensions of social support were ${\overline{\text{x}}}$ = 72.87 (SD = 15.12) for tangible support, ${\overline{\text{x}}}$ = 55.70 (SD = 16.01) for emotional/informational support, ${\overline{\text{x}}}$ = 52.65 (SD = 15.85) for positive social interaction, and ${\overline{\text{x}}}$ = 55.78 (SD = 17.35) for affectionate support. In terms of structural support, i.e., the average number of close friends and relatives that adult patients with LOPD feel at ease with and can talk to was 5.63. Patients with LOPD perceived that they received more tangible social support than other adult patients with RDs (${\overline{\text{x}}}$ = 72.9 vs. ${\overline{\text{x}}}$ = 64.8, *p* < 0.001). However, in the other three dimensions, the amount of perceived social support or structural support did not seem to differ significantly between the patients with LOPD and those with other RDs.

In terms of QOL of adult patients with LOPD, the average scores for the four dimensions were: ${\overline{\text{x}}}$ = 33.77 (SD = 18.28) for physical health, ${\overline{\text{x}}}$ = 43.81 (SD = 21.70) for psychological health, ${\overline{\text{x}}}$ = 39.43 (SD = 16.93) for environmental health, and ${\overline{\text{x}}}$ = 46.20 (SD = 19.76) for social relationships. Compared with other adult patients with RDs, the physical health QOL of people with LOPD was significantly lower (${\overline{\text{x}}}$ = 33.8 vs. ${\overline{\text{x}}}$ = 48.2, *p* < 0.001), whereas their QOL in the other three dimensions did not differ significantly from those of other patients with RDs.

### Univariate analysis

Table 2 presents the results of the univariate analysis of the four dimensions of QOL. At the level of *p* < 0.05, factors that significantly contributed to adult patients’ physical health QOL were age, reliance on assistive devices, and positive social interaction as an aspect of social support. As for psychological health QOL, the significant contributors were employment status, reliance on assistive devices, and positive social interaction. As for environmental health QOL, the significant contributors were age, employment status, reliance on assistive devices, and positive social interaction. As for social relationship QOL, the significant contributors were employment status, reliance on assistive devices, and positive social interaction.

**Table 2:** Univariate analysis of the four dimensions of QOL

	Physical health		Psychological health		Environmental health		Social relationships	
	Value^†^	*p* value^‡^	Value	*p* valve	Value	*p* value	Value	*p* value
Age	− 0.35	**0.003**	− 0.07	0.595	− 0.24	**0.047**	− 0.02	0.874
Gender								
Female	32.04(18.71)	0.411	42.94(20.7)	0.730	37.67(15.92)	0.372	47.22(19.72)	0.655
Male	35.71(17.87)		44.79(23.07)		41.41(18.05)		45.05(20.06)	
Education								
Primary school or lower	26.43(9.65)	0.467	22.5(20.33)	**0.037**	25(12.69)	0.125	36.67(22.52)	0.267
Middle school	30.95(19.78)		40(26.25)		38.75(14.55)		41.67(16.37)	
High school or above	35.42(18.46)		47.22(19.13)		41.15(17.5)		48.61(20.29)	
Employment								
No	30.03(17.57)	** < 0.001**	41.44(22.87)	**0.020**	37.27(17.82)	**0.004**	42.44(19.31)	**0.001**
Yes	48.21(13.42)		52.98(13.42)		47.77(9.37)		60.71(14.41)	
CHE								
No	37.32(18.74)	0.102	47.92(18.01)	0.340	42.5(15.75)	0.160	51.67(18.06)	0.142
Yes	29.62(16.14)		42.38(22.46)		36.43(15.56)		43.7(20.31)	
Rural/urban								
Urban	32.55(17.5)	0.477	44.7(18.9)	0.683	38.49(15.57)	0.568	44.7(20.26)	0.391
Rural	36.01(19.81)		42.19(26.45)		41.15(19.41)		48.96(18.93)	
Reliance on assistive devices								
None	54.76(18.16)	** < 0.001**	48.61(25.91)	**0.022**	55.73(25.88)	**0.006**	61.11(13.61)	**0.004**
Some	36.66(16.83)		50(18.89)		41.54(14.18)		50.74(18.73)	
A lot	25.77(15.71)		35.27(21.89)		33.37(15.45)		37.5(18.77)	
Medicated								
No	33.72(18.05)	0.958	43.78(22.59)	0.963	39.6(17.21)	0.797	46.86(20.19)	0.347
Yes	34.18(21.71)		44.05(12.47)		37.95(15.35)		40.48(15.54)	
Social support								
Tangible support	0.00	0.971	0.22	0.068	0.18	0.137	0.15	0.230
Emotional/informational support	0.19	0.119	0.49	** < 0.001**	0.25	**0.038**	0.51	** < 0.001**
Positive social interaction	0.37	**0.002**	0.55	** < 0.001**	0.41	**0.001**	0.60	** < 0.001**
Affectionate support	0.12	0.314	0.46	** < 0.001**	0.21	0.081	0.39	**0.001**
Structural support	0.09	0.459	0.35	**0.004**	0.20	0.109	0.36	**0.003**

Bold values indicate statistical significance was defined as* p* < 0.05

^†^For categorical variables, statistics on QOL are presented as mean (SD). For continuous variables, statistics on QOL were presented as correlation coefficients

^‡^For categorical variables, *p* values of the t-test or ANOVA results are shown. For continuous variables, the *p* values of the correlation test results are shown

### Multivariable analysis

Results from the multiple linear regression are presented in Table 3.

**Table 3:** Linear regression results of the four dimensions of QOL

	Physical health	Psychological health	Environmental health	Social relationships
Age	− **0.75 [**− **1.25, **− 0.24]**	**–**	− **0.79 [**− **1.27, **− 0.3]**	–
Education				
Primary school or lower	–	Reference	–	–
Middle school	–	− 4.07 [− 20.68, 12.53]	–	–
High school or above	–	− 4.3 [− 19.71, 11.11]	–	–
Employment				
No	Reference	Reference	Reference	Reference
Yes	7.98 [− 1.83, 17.8]	11.67 [1.47, 21.87]*	11.29 [2.4, 20.19]*	11.53 [1.96, 21.11]*
CHE				
No	Reference	–	–	–
Yes	− 6.48 [− 14.68, 1.73]	–	–	–
Reliance on assistive devices				
None	Reference	Reference	Reference	Reference
Some	− 10.58 [− 26.43, 5.28]	− **16.12 [**− **30.7, **− 1.55]*	− 12.08 [− 25.05, 0.88]	− **15.89 [**− **29.62, **− 2.17]*
A lot	− **17.8 [**− **34.24, **− 1.37]*	− **22.67 [**− **37.67, **− 7.66]**	− **17.8 [**− **31.21, **− 4.4]*	− **22.12 [**− **36.26, **− 7.98]**
Social support				
Tangible support	–	− 0.01 [− 0.31, 0.3]	–	–
Emotional/informational support	–	− 0.25 [− 0.8, 0.3]	− 0.29 [− 0.74, 0.16]	− 0.25 [− 0.73, 0.23]
Positive social interaction	0.28 [0.04, 0.51]*	0.71 [0.21, 1.21]**	0.72 [0.29, 1.15]**	0.7 [0.24, 1.16]**
Affectionate support	–	− 0.21 [− 0.54, 0.13]	− 0.22 [− 0.52, 0.07]	− 0.2 [− 0.52, 0.12]
Structural support	–	− 0.15 [− 0.86, 0.57]	0.16 [− 0.51, 0.82]	− 0.16 [− 0.85, 0.53]

Bold values indicate statistical significance was defined as* p* < 0.05

***p < 0.001; **p < 0.01; *p < 0.05

With increased age, the patients experienced a significant decrease in their physical health QOL (β = − 0.75, *p* < 0.01) and environmental health QOL (β = − 0.79, *p* < 0.01).

Educational level and CHE did not seem to correlate to different QOL scores in any dimension. Being employed is correlated with significantly higher scores in the domains of psychological health (β = 11.67, *p* < 0.05), environmental health (β = 11.29, *p* < 0.05), and social relationship QOL (β = 11.53, *p* < 0.05). Those who relied heavily on assistive devices had lower physical health (β = − 17.8, *p* < 0.05), lower psychological health (β = − 22.67, *p* < 0.05), lower environmental health (β = − 17.8, *p* < 0.05), and lower social relationship QOL (β = − 22.12, *p* < 0.01) than those who did not. Those who relied on assistive devices to some extent also had lower psychological health (β = − 16.12, *p* < 0.01) and lower social relationship QOL (β = − 15.89, *p* < 0.05) than those who did not. A one-unit increase in positive social interaction as a form of social support is correlated with a significant increase in physical health (β = 0.28, *p* < 0.05), psychological health (β = 0.71, *p* < 0.01), environmental health (β = 0.72, *p* < 0.01), and social relationships (β = 0.70, *p* < 0.01).

## Discussion

To the best of our knowledge, this study is the first to comprehensively explore the levels and factors associated with QOL among patients with LOPD in China. Compared to previous research that compared patients with PD with the healthy population, our study makes a significant contribution in that we took patients with other RDs as the comparison group. We believe that our choice of comparison group better highlights the problems faced by adult patients with LOPD, which are otherwise neglected by the existing health care and support systems in China. Another contribution of our study is that we focused on sub-dimensional scores rather than the total score of QOL, which can provide more specific suggestions for RD healthcare policymaking.

We found that adult patients with LOPD and with other RDs in China had similar QOL in the domains of psychological and environmental health and social relationships; however, the former suffered an even lower QOL in the domain of physical health than the latter. This is, to some extent, consistent with previous findings in the literature where patients with PD were compared with the general population [8, 37, 38]. This is due to the fact that the special pathological features of PD, which cause the accumulation of glycogen in the skeletal muscles, heart, liver, and nervous system, may substantially restrict the ability of patients with LOPD to move or breathe [37]. Our study notes that physical vulnerability may influence patients with LOPD more than patients with other RDs. This finding calls for more efforts to promote physical-health-related QOL among people with LOPD.

According to a recent review, PD has now been considered as a multisystem disorder [39]. While IOPD is characterized by cardiomyopathy, respiratory insufficiency and severe muscle hypotonia, LOPD may gradually progress to significant motor disability, respiratory insufficiency, and neuropsychological dysfunction [39, 40]. As the only standard of care that is available for patients with PD, ERT has proven to improve walking abilities [41] and respiratory function [42], and to reduce morbidity in adults with LOPD on long-term [43] or with early treatment [44]. For patients with IOPD, a recent study shows that ERT in combination with prophylactic immune tolerance induction initiated within the first month of life has yielded significant clinical benefits and survival rate in patients with CRIM-negative infantile PD, the most severe PD phenotype [45]. Hence, ERT can positively affect patients’ physical quality of life [11].

Meanwhile, as the study reveals, positive social interaction plays a very important role in increasing patients’ QOL across all domains. Positive social interaction was measured by three questions in the MOS-SSS instrument: How often is each of the following kinds of support available to you if you need it? (1) someone to have a good time with; (2) someone to get together with for relaxation; and (3) someone to do something enjoyable with. Hence, in general, positive social interaction in this study refers to the possibility and frequency for patients with LOPD to engage in social interactions for fun and relaxation [46]. Due to the deteriorating physical conditions suffered by patients, especially those without proper treatment, it is highly like that many patients with LOPD will gradually become disconnected with social life and lose positive social interaction with the world outside. Our study highlights the important role of positive social interaction in enhancing patients QOL, which can only be achieved by involving not only the patients and their families but also the community and the public. On the one hand, direct support provided by social workers or local non-government organizations to help patients and families address difficulties in daily living will greatly alleviate the caring burden and thus allow the main caregivers to allocate time and energy for the patients to have quality social life. On the other hand, elevating the awareness of the public on RDs in general and on PD in particular will eliminate misunderstandings and discriminations against patients with RDs, which will in turn remove the psychological barriers and stress that patients may have that prevent them from actively engaging with others [47]. By knowing the patients better, the public will also be mobilized to initiate more positive interactions with the patient community, hence. creating a more supportive social environment for patients with LOPD and other RDs.

No statistical differences were observed in the domains of psychological health, environmental health, and social relationships of QOL. Such findings are consistent with a previous study in Italian which found that patients with different RDs shared the same feeling of hopelessness and a sense of being marginalized that would strongly and negatively affect their self-image and life orientation [47]. The impact of RDs on psychological, environmental, and social QOL may be more closely related to social misconceptions and even stigma of RDs and patients with RDs than to the physiological nature of the diseases themselves [47]. Data from the Australian adolescent patients with RDs revealed that nearly half (46.4%) of the patients had ever experienced bullying victimization in school, and that many of their classmates (75.6%) and the teachers (53.7%) did not understand the child’s rare health condition [48]. Another study on patients with Alpha-1 antitrypsin deficiency (AATD) in the United States reported that the most memorable stigmatization experiences of the participants were rejection from family members, friends and romantic partners, followed by contempt from employers and health care providers [47]. Regardless of the type of patients with RDs, their perceptions of psychological stress, a sense of security from the environment, and social interactions with others could be similar. Recent research has emphasized that psychological adjustments are beneficial to patients’ well-being [49]. Yet more studies are needed to identify practical and effective ways for patients with LOPD and other RDs to improve their mental health.

ERT has reduced morbidity, but QOL is still impacted. Therefore it is important to identify ways to improve their QOL should be a core mission of patient-centered care. This study sought to contribute to this mission by exploring the contributors of patients’ QOL. Among all the potential contributors that we included in this study, gender did not seem to make a difference in terms of the four domains of QOL among the patients with LOPD in China. Age was negatively associated with the physical and environmental health domains of QOL, but it had no significant association with mental health or social relationships.

A systematic review among 1,214 patients with RDs in the United States noted that income level is positively associated with QOL in all domains [50]. Our study partly echoes these findings. On the one hand, we found that employment is significantly correlated with patients’ psychological, environmental, and social relationship QOL. This is reasonable because employed patients tend to perform better in their social roles and have more chances to interact with other people. Meanwhile, higher levels of QOL may also allow patients to have a higher employment rate. On the other hand, the association between employment and physical health were significant in univariate analysis but became non-significant in the multivariable model, implying a weak association between employment and patients’ physical health. It may imply that the association between employment and physical conditions might be mediated or moderated by other factors, such as positive social interaction. It is also possible that having a job may increase the patients’ subjective feelings of physical health. Therefore, policymakers and caregivers may need to think about how to create work opportunities that are suitable for these patients.

One surprising finding of our study is that there was no statistical difference between patients with LOPD and those with other RDs in terms of the proportion of patients suffering from CHE, even though ERT is recognized as one of the most expensive orphan drugs in the world. We think the most likely reason lies in the indicator of CHE itself. CHE is a binary indicator used for all populations and does not take into consideration disease-specific characteristics. Studies have indicated that RDs tend to pose a high economic burden on patients [51, 52]; in other words, the existing benchmark for judging CHE may be able to distinguish between rare and common diseases, but lacks sufficient sensitivity to distinguish between patients with PD and with other RDs. This inadequate sensitivity may also explain why nearly 70% of patients with RDs, which is much higher than the average proportion of CHE among the general population (12.9%) [53].

The problem faced by patients with PD, i.e., high healthcare expenditures, does not exist in China alone—in Europe, the average cost of ERT for PD is approximately EUR 300,000 (approximately RMB 2,360,000) per patient per year [1]. The financial burden of patients with PD in China can be even more devastating, because, at the time of the survey, almost all patients with PD must pay out-of-pocket. There is almost no insurance coverage from the government or the market to help alleviate the burden.

Another noteworthy finding of our study was that there is no significant association between CHE and the QOL in patients with LOPD, which is inconsistent with studies conducted in the United States and Europe [54, 55]. Although patients can have adverse infusion reactions, a systematic review of 21 international articles concluded that most adverse events were mild or moderate, and ERT is well tolerated in general [56]. Besides, reduced bodily pain, improved motor performance and ambulation status, and promoted patients’ quality of life in both physical and mental components was widely observed especially at the early stages of treatment. Hence, despite its high cost, ERT has been highlighted in some countries as a significant promoter of QOL [57]. Given the high price of ERT and the nearly complete lack of health insurance coverage on it in China at the time of this survey, a higher CHE may indicate the utilization of ERT or other forms of treatment, which should have potentially helped, at least, increase the patients’ physical health. However, this is not what we found in our study. Although no existing literature could provide explanation to such a finding, based our knowledge on the community of patients with LOPD in China, the majority of the patients who received ERT used the medicine neither continuously nor with sufficient doses. As a result, the effectiveness of the treatment was quite limited.

This study found that a higher level of reliance on assistive devices was negatively associated with all four dimensions of QOL. A large-scale study using the International Pompe Survey also revealed that wheelchair- and ventilator-dependent patients were more likely to report lower health-related QOL in terms of physical functioning, physical role functioning, and social functioning than the general population [8]. Yet in the same study, functional disability was not found to be related to mental health-related QOL. However, our study found otherwise; that is, the influence of functional disability was pervasive on QOL in patients with LOPD. This may be because patients who rely heavily on assistive devices are less likely to go outdoors and have fewer chances to interact with others. More attention should be paid to these socially isolated patients. Future research should explore the impact of an active lifestyle, especially among patients who require assistive devices. The findings should be tested to determine whether they can be generalized to the global context.

Previous studies on QOL in patients with LOPD mostly focused on the financial and physical burdens presented by the disease; however, the humanistic aspect was largely overlooked. Dekker et al. indicated that the painful processes of learning, coping, and adapting to disease-related psychology could induce a humanistic burden on the social capital and social support of the patients and their caregivers [10]. Our findings indicated that promoting positive, successful interactions should be emphasized in improving QOL in patients with LOPD beyond the conventional emphasis on medication. Patient organizations may consider providing opportunities for patients to talk with their peers and share good news with friends in their programs. This study also found that unlike social interaction, tangible emotional/informational and affectionate social support might not be associated with patients’ QOL. It is worthy of note that one review of the spiritual needs of chronic disease patients emphasized that the feeling of being in charge or being helpful was more effective in improving patients’ psychological resilience than the feeling of being weak or being helped [58]. Therefore, when promoting social interactions, QOL promotional programs and patient organizations may try to offer more opportunities for reciprocal interactions rather than treating the patients as recipients alone.

### Limitations

There are a few limitations to this study. First, this is a self-response cross-sectional survey that involves a rather small sample size. The inference made from this sample needs to be carefully examined. Second, the data were collected by an online survey. Although this approach could maximize the coverage of patients residing sporadically over a large country, whether or not the subjects would be able to understand the questions correctly or to provide authentic answers are of concern. Third, we did not include other possible factors associated with LOPD or QOL, such as comorbidities, clinical symptoms, and accessibility to medical resources. Fourth, there is neither a specific QOL scale for patients with RDs nor for patients with LOPD in particular. Therefore, we used the WHOQOL-BREF, which consists of abstract questions that are not suitable for children. Hence, non-adult patients with LOPD or IOPD were excluded from our study. Further relevant studies are warranted.

## Conclusion

This study comprehensively explored the levels of and contributors to the QOL of adult patients with LOPD in China. Measured by the WHOQOL-BREF, the average physical health QOL for this group of patients was very low. However, social interactions, especially those realized via employment, were found to play an important role in QOL in patients with LOPD in almost all dimensions. Compared with offering tangible, informational, or affectionate social support, it might be more important to encourage patients with LOPD to be socially active and to help them better included in social life. Additionally, most Chinese patients with LOPD cannot afford ERT due to inadequate financial support. They inevitably become disabled at an earlier age, which will, in turn, constrain their social life and decrease their QOL. Hence, how to provide adult patients with LOPD equal or even more opportunities to be socially engaged is something that healthcare providers and policymakers need to consider.

## Data Availability

Not applicable.

## Abbreviations

CHE: Catastrophic health expenditure
ERT: Enzyme replacement thrapy
IOPD: Infantile onset Pompe disease
LOPD: Late onset Pompe disease
MOS-SSS-CM: Chinese Mandarin version of the Medical Outcomes Study Social Support Survey
PD: Pompe disease
QOL: Quality of life
WHOQOL-BREF: World Health Organization Quality of Life: Brief Version
